# Translating the Biology of Aging into New Therapeutics for Alzheimer’s Disease: Senolytics

**DOI:** 10.14283/jpad.2023.104

**Published:** 2023

**Authors:** M. Riessland, M.E. Orr

**Affiliations:** 1 Department of Neurobiology and Behavior, Stony Brook University, Stony Brook, NY, USA; 2 Center for Nervous System Disorders, Stony Brook University, Stony Brook, NY, USA; 3 Department of Internal Medicine Section on Gerontology and Geriatric Medicine, Wake Forest University School of Medicine, Winston-Salem, NC, USA; 4 Salisbury VA Medical Center, Salisbury, NC, 28144, USA.

**Keywords:** Alzheimer’s disease, biology of aging, cellular senescence, senolytics, tau

## Abstract

The recent FDA-approval for amyloid lowering therapies reflects an unwavering commitment from the Alzheimer’s disease (AD) research community to identify treatments for this leading cause of dementia. The clinical benefits achieved by reducing amyloid, though modest, provide evidence that disease modification is possible. Expanding the same tenacity to interventions targeting upstream drivers of AD pathogenesis could significantly impact the disease course. Advanced age is the greatest risk factor for developing AD. Interventions targeting biological aging offer the possibility of disrupting a foundational cause of AD. Senescent cells accumulate with age and contribute to inflammation and age-related diseases like AD. Senolytic drugs that clear senescent cells improve healthy aging, halt AD disease progression in animal models and are undergoing clinical testing. This review explores the biology of aging, the role of senescent cells in AD pathology, and various senotherapeutic approaches such as senolytics, dampening the SASP (senescence associated secretory phenotype), senescence pathway inhibition, vaccines, and prodrugs. We highlight ongoing clinical trials evaluating the safety and efficacy of the most advanced senolytic approach, dasatinib and quercetin (D+Q), including an ongoing Phase II senolytic trial supported by the Alzheimer’s Drug Discovery Foundation (ADDF). Challenges in the field of senotherapy for AD, including target engagement and biomarker development, are addressed. Ultimately, this research pursuit may lead to an effective treatment for AD and provide the field with another disease-modifying therapy to be used, alone or in combination, with other emerging treatment options.

## Introduction: Biology of Aging and Alzheimer’s disease

Older adults fear developing dementia more than any other health condition (1–3). The World Alzheimer Report (2021) estimates that over 55 million individuals live with dementia (4); Alzheimer’s disease (AD) is the leading cause. AD is histologically defined by the accumulation of Aβ plaques (extracellular) and tau protein aggregates (intracellular) in the brain (5). Clinical trials that successfully reduce these abnormal protein deposits have demonstrated only modest disease-modifying effects (6, 7) highlighting the continued need for additional therapeutic approaches to prevent, slow and treat AD.

Historically, research focused on identifying AD drug targets and interventions have experienced significant failures with a ~1% success rate (8). One major hurdle in drug development is an incomplete understanding of the underlying mechanisms that drive Aβ plaques and tau-containing neurofibrillary tangles (NFTs) in the brain which may begin decades prior to cognitive decline (9–11). The greatest risk factor for developing AD is advanced age (12). While chronological aging is inevitable, research using laboratory organisms has identified cellular and molecular processes that directly influence successful biological aging (13). These “hallmarks of aging” satisfy three conditions: (i) they become apparent with advancing age, (ii) experimentally perturbing them causes poor aging (iii) therapeutically intervening upon them prevents, delays or reverses aging phenotypes.

Initially identified as nine fundamental biological drivers of aging (13), the list has been updated to include twelve hallmarks: genomic instability, telomere attrition, epigenetic alterations, loss of proteostasis, disabled macroautophagy, deregulated nutrient-sensing, mitochondrial dysfunction, cellular senescence, stem cell exhaustion, altered intercellular communication, chronic inflammation, and dysbiosis (14). Their organization into broad categories represents an interconnected hierarchy where the primary hallmarks reflect macromolecular damage (i.e., genome, proteome, organelles) which lead to antagonistic hallmarks, e.g., response to the damage. Integrative hallmarks refer to the downstream consequences of the persistent, irreversible stress.

Experimentally manipulating aging biology influences AD pathogenesis which provides a mechanistic link between increasing age and elevated risk of developing AD (reviewed, (15)). Moreover, many proposed AD hypotheses overlap with biology of aging hallmarks. For example, the amyloid hypothesis (16, 17) aligns with the decline in proteostasis (a primary hallmark); the cholinergic hypothesis (18) reflects deficits in intercellular communication; the mitochondrial cascade hypothesis (19) falls under an antagonistic hallmark; while the inflammatory hypothesis (20) represents one of the integrative hallmarks. Given the interconnected relationship among biological aging hallmarks, manipulating one has potential to impact others. For example, cellular senescence, classified as an antagonistic/secondary hallmark of aging, results as a consequence of persistent DNA and/or proteomic damage (primary hallmarks) that drives integrative hallmarks such as chronic inflammation and altered intracellular signaling, which further perpetuate other aging hallmarks. Targeting cellular senescence has emerged as an especially appealing therapeutic target given its contribution to persistent inflammation and functional decline across tissues and organ systems (reviewed, (21)).

## Cellular Senescence

The cellular senescence fate is initiated and sustained through an elaborate cellular stress response. Instead of reacting to stressors by uncontrolled proliferation (cancer) or apoptosis (degeneration), senescent cells survive in a stable cell cycle arrest. They undergo profound chromatin changes, metabolic alterations, cellular remodeling, and resistance to apoptosis (22–28). This switch in cell fate may be advantageous in tissues with limited replicative potential, like neurons in the brain and myocytes of the heart; however, senescent cells infamously secrete high levels of pro-inflammatory cytokines, chemokines, and extracellular matrix-degrading proteins that negatively impact the extracellular environment and induce spread of senescence to non-senescent cells (29–32). This toxic secretome, referred to as senescence-associated secretory phenotype (SASP), contributes to tissue destruction and chronic cell death.

**Figure 1.**
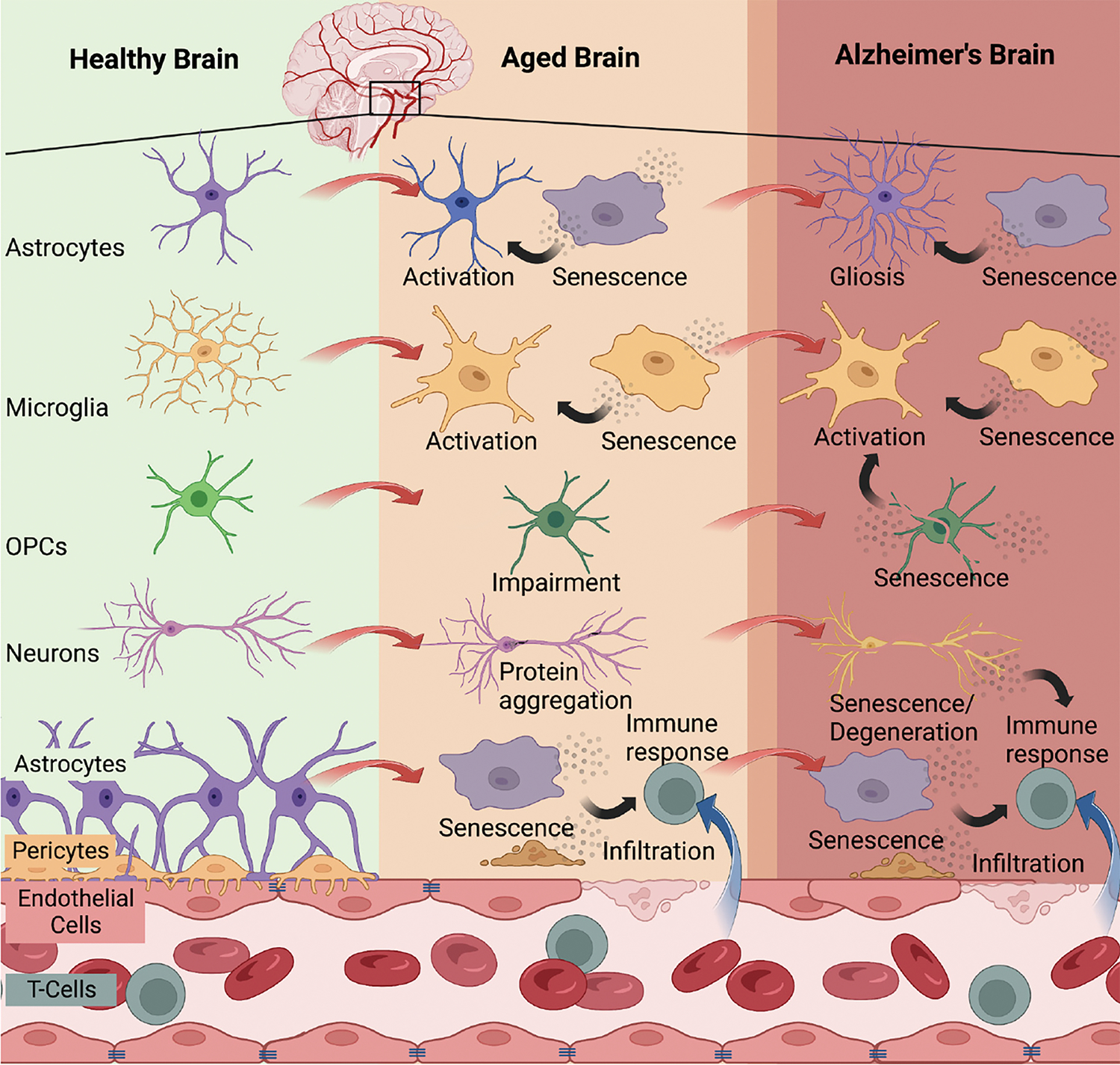
Brain cell senescence increases with advanced age and contributes to Alzheimer’s disease Multiple brain cell types have been identified to enter cellular senescence with advanced aging and/or AD including astrocytes (38), microglia (40), OPCs (37), neurons (35, 39), endothelial cells (41), and smooth muscle vascular cells (42). The accumulation of senescent cells with age leads to immune reactions that have detrimental effects on the brain. Persistent senescent cells can propagate the senescent phenotype, resulting in blood-brain barrier breakdown, infiltration of systemic immune cells, neurodegeneration, and gliosis, all of which are observed in the context of AD. Created with BioRender.com (Agreement no: ED25XPZLUF).

Preclinical studies indicate that senescent cell burden is low in young rodents and increases with chronological age in several tissues (33, 34). Tau (35, 36) and Aβ (37) accumulation induces premature cellular senescence in transgenic mouse models of AD neuropathology. Similarly, various senescent cell types have been identified in postmortem AD brain tissue including astrocytes (38), neurons (35, 39), microglia (40), oligodendrocyte precursor cells (37), and endothelial cells (41). We analyzed single-cell transcriptomic data generated from the dorsolateral prefrontal cortex of individuals with various levels of AD neuropathology; approximately 2% of brain cells were senescent, with excitatory neurons as the main contributing cell type (39). The cell cycle inhibitor, *CDKN2D*/p19^INK4d^, and NFT molecular signatures significantly overlapped with the senescent neuron population (39). Interestingly, bioinformatic analyses conducted on bulk tissue data obtained from healthy human brain tissue donors revealed that endothelial cells and microglia were the predominant senescent cell types, driven by *CDKN1A*/p21^WAF1^/CIP1 (42) revealing potential differences in senescent cell types between healthy and diseased states. Nevertheless, data generated from multiple groups have demonstrated the presence of senescent cells in postmortem human brain (Figure 1).

Proof of principle experiments utilizing genetically engineered progeroid mouse models conclusively demonstrated that senescent cells contribute to poor aging (43). Genetic ablation of senescent cells ameliorated the progeroid phenotypes and produced overall health benefits. A later study using the same genetic approach to clear senescent cells, but this time in naturally aging mice, provided further evidence that removing senescent cells improves healthy aging (44). The discovery of compounds that selectively remove senescent cells eliminated the need for genetic models and catapulted this field of study (24, 45). The initial discovery of senolytics involved bioinformatics and transcriptomics to evaluate RNA and protein expression profiles of senescent cells. The approach revealed that the anti-apoptotic survival of senescent cells relied on six senescent-cell anti-apoptotic pathways (SCAPs) (45, 46). These pathways include BCL-XL, the cell cycle inhibitor p21, the ephrins EFNB1 and EFNB3, serpin PAI1, the oxygen sensor HIF-1α and phosphatidylinositol 3-kinase-delta (PI3Kδ). Decreasing the expression of key proteins in SCAP pathways induced senescent cell death, promoting the discovery of small molecules and compounds that target these SCAPs, referred to as “senolytics”.

### Senolytics

Among the first senolytic agents discovered was a combination therapy consisting of Dasatinib (D; Sprycel^®^, Bristol Myers Squibb), a tyrosine kinase inhibitor that is FDA approved for use in humans for the treatment of leukemias, and Quercetin (Q), a flavonoid (45). Despite its ability to induce cancer cell apoptosis, D did not show strong capacity to clear senescent cells when used as a monotherapy. Similarly, the naturally occurring flavonoid Q possesses various biological activities such anti-inflammatory and relatively unspecific kinase inhibitory properties, but as a stand-alone-treatment displays only marginal senolytic effects. The D+Q combination targets a greater number of SCAPs (45), leading to effective senescent cell clearance across tissues and positively impacts several age-associated conditions and diseases. These include neurodegeneration, frailty, osteoporosis, vasomotor dysfunction, hepatic steatosis, insulin resistance, pulmonary fibrosis, chronic kidney disease, and skeletal muscle dysfunction (47).

#### Natural compounds

Similar to Q, fisetin is a naturally occurring flavonoid that is present in various fruits, vegetables, and teas (48, 49). It is widely used as a supplement and possesses diverse pharmacological effects, including antioxidant, antidiabetic, anti-inflammatory, anti-cancer, antibacterial, antiviral, and neuroprotective activities (48–50). On the molecular level, fisetin acts on multiple signaling pathways, including BCL-2, PI3K/AKT, p53, and NF-κB, all of which are critical for senescence. Consequently, research has demonstrated that fisetin exhibits senolytic activity, which was discovered in a screen of flavonoid polyphenols in senescent human and murine fibroblasts (51, 52). It has been shown that fisetin reduced senescence in a cell type-specific manner: in white adipose tissue (WAT), it reduced mesenchymal stem/progenitor, immune, and endothelial cells, while senescent dendritic cells and macrophages were not changed. Interestingly, fisetin has been reported to restore tissue homeostasis, reduce age-related pathology, and extended median and maximum lifespan in wild-type mice (51). In the context of AD, fisetin has been shown to modulate inflammatory pathways and maintain cognitive function in AD transgenic mice (53) and to enhance memory and induce hippocampal long-term potentiation in rats (54). These findings suggest that fisetin can cross the blood brain barrier and that it has beneficial effects on the hippocampus and can ameliorate AD symptoms *in vivo.* Given the positive results across tissues and diseases of aging, fisetin has entered clinical testing. Currently around 20 clinical trials are ongoing using fisetin. These clinical trials target diverse age-related diseases including osteoarthritis (NCT05276895), frailty (NCT03675724), mild cognitive impairment (NCT02741804), aging (NCT04994561) and others (55).

In addition to fisetin and Q, other natural substances derived from plants also exhibit senolytic properties, such as piperlongumine (PL) and its analogues (56), or the curcumin analog EF24 (57). Both compounds have been shown to ablate senescent cells in a ROS (reactive oxygen species)-independent manner and are able to synergize with other senolytic compounds like navitoclax (ABT-263) (56, 58, 59). These natural senolytic compounds offer potential therapeutic strategies for combating age-related diseases and improving health-span. Due to the lack of comprehensive studies on natural compounds, little is known about their dosing and side effect profiles. The initial clinical studies on fisetin, and others, will be critical to inform the field on the safe use of natural compounds as senolytic therapies.

#### BCL-2 family inhibitors

Senescent cells enter a near-permanent cell cycle arrest and activate an anti-apoptotic program to persist in the tissue where they contribute to inflammation. An important anti-apoptotic pathway that that allows senescent cell survival involves expression of members of the BCL-2 family of proteins, including BCL-2, BCL-XL, and BCL-W (60). Inhibition of the BCL-2 pathway induced apoptosis of some senescent cell types, such as mouse and human fibroblasts, HUVECs, as well as cells of the lung and epidermis, but not primary human preadipocytes (60, 61). Consequently, BCL-2 family inhibitors have been identified to function as senolytics, including ABT-737, ABT-263 (Navitoclax), A-1331852, and A-1155463. The first characterization of ABT-737, an inhibitor of BCL-W, BCL-XL, and BCL-2, has been shown to specifically induce apoptosis in senescent cells in a BCL family-dependent manner (60). In mice, ABT-737 efficiently removed senescent cells in the lungs as well as in the epidermis, leading to an increased hair-follicle stem cell proliferation (60). However, ABT-737 has limited oral bioavailability and aqueous solubility. To overcome these limitations, ABT-263 (Navitoclax), a derivative of ABT-737, was developed as an orally bioavailable panBCL inhibitor (62). In addition to its *in vitro* senolytic activity, ABT-263 has been shown to rejuvenate aged hematopoietic stem cells and effectively clear senescent cells in irradiated or aged mice (63). Unfortunately, significant side effects such as thrombocytopenia and transient thrombocytopathy have been reported for ABT-263 (64). In addition to systemic adverse effects, inhibiting BCL-2 family members could be problematic for diseases and conditions of the CNS. In our *in vitro* model, we found that treatment with ABT-737 is toxic to stem cell derived dopaminergic neurons (65). The underlying reason for this toxicity is most likely because neuronal development and survival is based on BCL-XL activity (66). Additionally, navitoclax is almost impenetrable to the blood brain barrier, thus, the current drugs are not able to reach the brain parenchyma (67). Thus, despite the demonstrated senolytic effects of BCL-2 family inhibitors, their clinical translation is limited due to potential side effects, off-target effects on platelets and poor central nervous system (CNS) penetrance. Further research is needed to develop more specific, safe and brain penetrant BCL-2 family inhibitors for senolytic therapies.

#### P53 pathway modulators

P53 controls senescence and apoptosis in part via regulation of BCL-2 (68, 69). In line with this, p53 modulators have been investigated as potential senolytic agents. Forkhead box protein O4 (FOXO4) is an anti-apoptotic transcription factor which is upregulated in some senescent cells and prevents cell death by sequestering the nuclear protein p53 (70). A senolytic peptide called FOXO4-D-Retro-Inverso (FOXO4-DRI) has been reported to disrupt the interaction between FOXO4 and p53, allowing p53 to translocate to the cytosol and induce apoptosis. FOXO4-DRI selectively removed senescent cells and has shown benefits in alleviating age-related symptoms in animal models, including hypogonadism, skin phenotypes, fur density, and renal function.

Protein levels of p53 are regulated through its ubiquitination by the E3 ubiquitin ligase, murine double minute 2 (MDM2). Inhibitors of the MDM2/p53 interaction, such as UBX0101 and RG7112 (RO5045337), and inhibition of the de-ubiquitinating ubiquitin-specific peptidase 7 (USP7) increase and stabilize p53 levels and have been investigated for their senolytic effects. RG7112 selectively cleared senescent intervertebral disc cells, reduced senescence-associated secretory phenotype (SASP) factors, and improved disc matrix homeostasis in human disc tissue. UBX0101 induced apoptosis in senescent chondrocytes in an osteoarthritis mouse model. USP7 inhibitors, such as P5091 and P22077, selectively ablated senescent cells by upregulating p53 (71).

While p53 modulators, including FOXO4-DRI, MDM2 inhibitors, and USP7 inhibitors have shown senolytic effects by selectively eliminating senescent cells or promoting their apoptosis in the laboratory, translating these findings to the clinic have so far been unsuccessful. A Phase II clinical study of UBX0101 for osteoarthritis did not show statistical significance compared to placebo. Given that the role of p53 in senescence regulation is complex, with both promoting and inhibitory effects depending on cell type and stress levels, manipulating the levels of p53 could have unwanted consequences. For example, activity of p53 can induce p21-dependent senescence. Moreover, some agents that have been used to interfere with p53 function are peptide-based, which would render them difficult to apply for brain senescence. In summary, additional research is needed to understand the intricate molecular mechanisms of p53 in regulating senescence in order to optimize the efficacy and safety for clinical applications.

#### Immune system modulation for senolysis

Using senescence immune surveillance, senescent cells are naturally removed by the body’s own immune cells such as macrophages, T-cells, natural killer cells (NK cells) (72) and microglia in the brain (73, 74). However, due to age-related immunosenescence and the capability of senescent cells to evade immune surveillance, senescent cells are spared and accumulate in diverse tissues (75). Consequently, increasing the efficiency of immune surveillance has become a novel strategy to target senescent cells. One strategy is the use of so-called chimeric antigen receptor T cells (CAR-T cells) which have been developed for cancer treatment (76). In brief, T cells derived from patients are primed by genetic manipulation to target and remove cells that express a specific antigen. Using urokinase-type plasminogen activator receptor (uPAR) as antigen, it has recently been shown that CAR-T cells can actively remove senescent cells and ameliorate diverse pathologies in mouse models (77). Similarly, an antibody-based approach to target senescent cell surface protein DPP4 has been utilized to ablate senescent cells *in vitro* (78). Moreover, an anti-senescence vaccine-like protocol has been recently reported. This approach focused on glycoprotein nonmetastatic melanoma protein B (GPNMB) as a molecular target for senolytic therapy. Suda et. al. showed immunization of mice against Gpnmb resulted in a reduction of Gpnmb-positive cells. Furthermore, it was shown that senolytic vaccination improved normal and pathological phenotypes associated with aging, and extended the male lifespan of progeroid mice (79).

The concept of using CAR-T cells as senolytic agents is still in its early stages, and research in this area is ongoing. Since immune cell trafficking into the CNS is tightly regulated by the blood brain barrier which selectively only allows entry of immune cell subsets required for immune surveillance (80), it remains unknown if CAR-T would efficiently reach senescent cells in the brain parenchyma. One additional challenge is identifying specific and reliable markers that are exclusively expressed on senescent cells to ensure accurate targeting by CAR-T cells or antibodies or to use for immunization. Moreover, immune system-based approaches remove senescent cells by an inflammatory response, which could be problematic in the context of AD where neuroinflammation is present. While harnessing the immune system offers promise to clear senescent cells, the potential impact of off-target effects and unintended immune responses needs to be carefully evaluated to minimize potential harm to healthy tissues.

### Senomorphics

An alternative approach of the use of senolytics, which clear senescent cells, is the use of “senomorphics” to ameliorate their SASP (summarized in: (55)). This approach may be especially favorable when difficult to replace cells, like neurons in the brain, become senescent. It remains unknown whether senescent neurons, or other cells with a similar gene expression profile, should be actively removed from its tissue or if it would be advantageous to simply target their detrimental SASP and ameliorate the likelihood of senescence, and associated inflammation, spreading. Senomorphics are currently under development to lower this inflammatory burden. Numerous signaling pathways, such as cyclooxygenase 2 (COX-2), mammalian target of rapamycin (mTOR), mitogen-activated protein kinase (MAPK) signaling, phosphoinositide 3 kinase (PI3K), and the GATA4/p62-mediated autophagy pathways result in the stimulation of NF-κB and/or C/EBPβ pathways, which in turn regulate the SASP secretome (81, 82). This convergence renders the NF-κB and C/EBPβ pathways promising drug targets for the modulation of the SASP. Accordingly, a multitude of molecules and antibodies to interact either with NF-κB and C/EBPβ transcriptional activities at different levels have been developed (summarized in: 81).

#### mTOR inhibition

Rapamycin is a well-studied FDA approved immunosuppressive drug that has been shown to extend lifespan in yeast, worms, flies, and mice (83, 84). Furthermore, it has been reported to reduce cellular senescence and suppress the SASP. Rapamycin functions by inhibiting mTOR to stimulate autophagy, among other pathways. Even though a promising candidate for lifespan extension and SASP repression, multiple side effects including metabolic dysregulation, impaired wound healing, and hyperlipidemia have been reported (85). Given the positive effects in multiple animal models of aging and neurodegeneration, including models of AD, clinical trials for rapamycin in AD are underway (NCT04200911, NCT04629495).

#### NF-κB inhibition

One of the most central transcription factors for the regulation of immune responses and the regulation of the SASP is NF-κB. Genetically enhancing NF-κB activity causes neuroinflammation and increases senescent cell burden in the brain (86). In a basal state, the function of NF-κB is repressed by IκB inhibitors. During stress- or inflammatory-responses, IκB inhibitors are phosphorylated by the so-called IKK complex (87). Interestingly, inhibition of the binding to IKK by using a peptide inhibitor reduced NF-kB activity and thereby senescence in progeroid mice (88). Consequently, a small molecule was developed to inhibit the interaction between NF-κB and IKK. This molecule, SR12343, significantly reduced the release of SASP factors, senescence, and improved markers of senescence in progeroid and naturally aged mice (89). The inhibitory molecule SR12343 has been shown to cross the blood brain barrier positioning it as an interesting compound to test in the context of AD and other neurodegenerative/neurological conditions (90). However, given that NF-κB is involved in diverse biological processes related to inflammatory response, cell adhesion, growth control, and protection against apoptosis (87), its inhibition could lead to multiple unwanted side effects. It will require thorough characterization before it can be applied in humans. Of note, the treatment of mice with the nonsteroidal anti-inflammatory drug, ibuprofen, mitigated senescent cell accumulation and neuroinflammation and improved cognitive function (86). However, clinical trials using ibuprofen have not successfully modified AD disease progression or outcomes (91, 92), which may reflect a need for combination therapies to target multiple aspects of this complex disease.

#### P38MAPK inhibition

The p38MAPK inhibitor SB203580, by decreasing the transcriptional activity of NF-κB, has been shown to reduce the levels of mRNA of genes of SASP factors in human senescent cells (81). Similarly, next-generation p38MAPK inhibitors UR-13756 and BIRB 796 inhibited IL-6 expression in human senescent fibroblasts, demonstrating their effectiveness in reducing SASP-related effects (93). Interestingly, inhibition of JAK/STAT signaling by ruxolitinib suppressed C/EBPβ transcriptional activity, leading to a reduction in systemic inflammation by SASP repression and improvement in fitness in elderly mice (94). Reducing the activity of the stress kinase p38MAPK may be particularly beneficial for the neuroinflammation common to AD. Because of this interest, a brain permeable and orally available p38MAPK inhibitor has recently been developed (MW150, a.k.a. MW01-18-150SRM) and is currently in a clinical trial for AD (NCT05194163).

In summary, several approaches show promise as senolytic therapies. The combination of D+Q effectively clears senescent cells and positively impacts age-associated conditions and diseases. Natural compounds like fisetin have also exhibited senolytic activity and are being tested in clinical trials. BCL-2 family inhibitors, such as ABT-263 (Navitoclax), induce apoptosis in senescent cells but have limitations regarding side effects and CNS penetrance. Modulators of the p53 pathway, such as FOXO4-DRI and MDM2 inhibitors, selectively eliminate senescent cells but face challenges in clinical translation. Immune system modulation shows potential for enhancing immune surveillance and clearing senescent cells, but is in early stages. While several senolytic and senomorphic therapies show promise in clearing senescent cells and impacting age-related conditions, many lack data on CNS penetrance and efficacy in AD relevant systems. Among these approaches, the first-generation D+Q combination has accumulated the most experimental data regarding safety and efficacy, and CNS bioavailability (95–99). Currently, D+Q represents the most mature, and promising, senolytic therapy for CNS conditions.

### Senolytics Improve AD Outcomes in Preclinical Studies

Multiple independent research groups have demonstrated that D+Q clear senescent cells across biological systems (model organisms), diseases and tissues. Senolytic target engagement does not require continuous receptor binding, enzyme modulation, or persistent modulation of biochemical pathways. Instead their mechanism of action is to temporarily disrupt anti-apoptotic pathways utilized by senescent cells for their survival. Moreover, senescent cells do not divide and accumulate slowly; as such, senolytics do not require continuous presence in the circulation. Preclinical studies in rodents have validated this approach; intermittent treatment with senolytics have conclusively reduced senescent cell burden in peripheral tissues (fat, lung, kidney, pancreas, cartilage, and muscle), improved pathologies in these tissues, and extended healthspan and lifespan (45, 46, 100–103).

We began exploring the efficacy of senolytics in tauopathy mouse models to determine the impact of senescent cells on brain structure and function relevant to AD neuropathology (35). In the study, we utilized four distinct tau transgenic mouse models including an aggressive, early age onset tauopathy (rTg(tau_P301L_)4510) *Mapt*^0/0^; late-onset slowly progressive tauopathy (rTg(tau_WT_)21221); tau knockout mice (*Mapt*^*0/0*^); mice with a combination of Aβ and NFT pathology (3xTgAD); and age-matched wild type mice. Additionally, we analyzed postmortem human brain tissue with histopathologically confirmed AD or progressive supranuclear palsy (PSP), an age-associated tauopathy that clinically manifests as parkinsonism with additional motor abnormalities and cognitive dysfunction, and is neuropathologically defined by NFTs, gliosis and neurodegeneration (104). Among the distinct tau transgenic mouse models, senescence was a robust and invariant response to tau accumulation that correlated with brain atrophy in mice and human PSP (35). Robust markers of cellular senescence including *Cdkn2a*, *Cdkn1a* and SASP (i.e., *Tnfa*, *Il1b*, *Clxcl1*, etc.,); tissue remodeling; and karyomegaly were observed in tau transgenic mouse brains (35). Transcriptomic analyses of laser capture microdissected neurons from postmortem human AD revealed that NFT-containing neurons expressed signaling pathways consistent with cellular senescence, including SCAPs, upreguled pro-survival and inflammatory pathways and downregulated cell death pathways (35).

**Figure 2.**
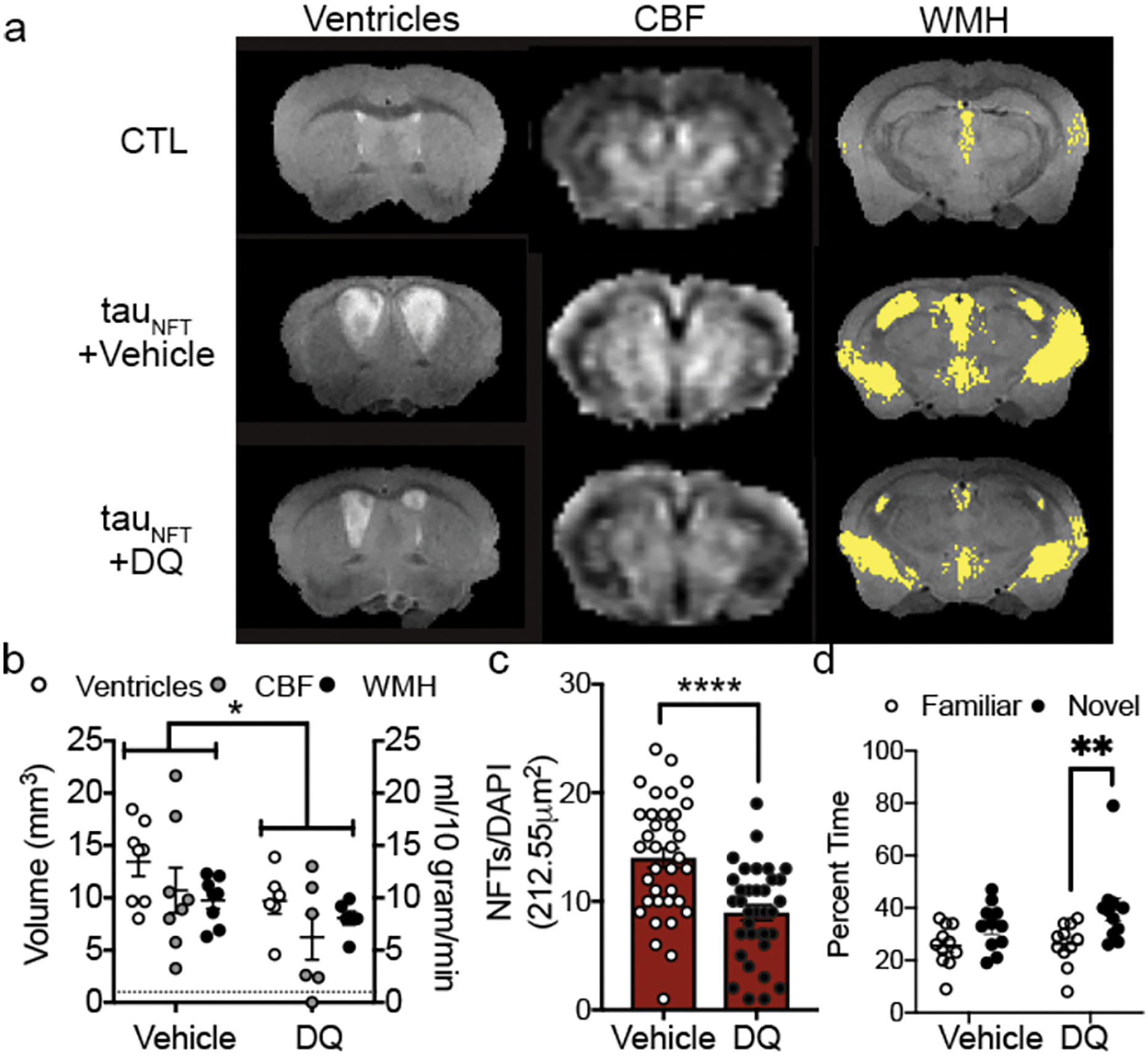
Senescent cell removal with D+Q improved brain structure and function in AD mice (a) MRI on advanced aged control (CTL) and transgenic (tau_NFT_) mice revealed abnormal ventricles, cerebral blood flow (CBF) and white matter hyperintensity (WMH) pathologies that were mitigated by (b) treatment with DQ. Two-way ANOVA, significant treatment effect p<0.05. (c) Quantification of the number of senescence neurons with NFTs between vehicle and DQ treated mice. Two-tailed t-test, **** p < 0.0001. (d) Cognitive behavior was assessed by time spent in familiar or novel arm of the y-maze. Two-way ANOVA, Sidak post-hoc. **: p = 0.0083. n=11/group. (Adopted from (122)).

To determine a causal role between senescent cells and tauopathy, we administered intermittent (once every two weeks) senolytic therapy (D+Q) to tau transgenic mice between the ages of 20–23 months old with already present tau pathology, neurodegeneration, inflammation and brain atrophy (35). The goal was to determine if senolytic therapy could effectively clear senescent cells in the CNS and thereby stop the progression of tau-associated pathogenesis in advanced disease stages. After three months of cyclic senescent cell removal using D+Q, we assessed cerebral blood flow, brain volume and ventricle volume with MRI to determine if removing senescent cells produced global effects on brain function and structure. D+Q treatment mitigated pathogenic ventricle enlargement, aberrant cerebral blood flow and white matter hyperintensity pathology (Figure 2a–c, adapted from (21)). We repeated these experiments in a separate cohort of tau transgenic mice. Behavior analyses indicated that D+Q treatment improved tau transgenic mouse discrimination index in the y-maze, providing additional evidence for improved brain function with D+Q treatment (Figure 2d).

Target engagement was confirmed by decreased phospho-tyrosine and NFT burden. We found that the improvements were mechanistically attributed to a significant reduction in NFTs and inflammation. We predicted that off-target effects of D+Q on non-senescent neurons would be evidenced by a decrease in total neuron density; however, we observed an overall increase in neuronal protein expression (e.g., NeuN, synaptophysin and PSD95). Combined with the D+Q associated reduction in ventricle size as assessed by MRI, a surrogate measure of neurodegeneration, the data suggested that clearing senescent cells was beneficial for neuronal preservation. Though we did not observe differences in total astrocyte or myelin protein expression, we noted an increase in Iba1, reflective of increased microglia. In peripheral tissue, senescent cells are cleared by macrophages. Microglia are the resident macrophage cells of the brain. Our interpretation was that elevated Iba1 may have reflected an increase in microglia-mediated clearance of cellular debris after senolytic treatment (35). Data from Dr. Maria Grazia Spillantini’s laboratory reported that microglia engulf NFT-bearing neurons that display phosphatidylserine on their cells surface (73). Subsequent studies by the same research team found that microglia develop a senescence-like phenotype after engulfing NFT-containing neurons, and they release toxic forms of partially digested tau (74). Given the downstream consequences of senescent cells, even when efficiently cleared by microglia, eliminating them with therapeutic interventions may potentially mitigate their abundance, reduce inflammation, and alter disease progression.

Collectively, work across multiple independent laboratories have reported on both amyloid and tau transgenic AD mouse models, postmortem human brain tissue from two distinct tau-expressing neurodegenerative conditions, and three senolytic compounds (Table 1). The results convincingly demonstrate that protein aggregation induces senescence of many cell types in the brain, and that senolytics effectively reduce pathogenesis (35–37). Experimental evidences indicate that neurons are a primary source of senescence in AD. While therapeutically clearing senescent neurons may not be an appealing approach, the brain’s immune system is already attempting to rid these harmful cells. Once the microglia engulf the senescent neurons, they become senescent, dysfunctional and amplify pathogenesis through inflammation, releasing toxic tau and failure to survey the brain microenvironment. In this way, therapeutically clearing senescent cells, even neurons, may prove beneficial as supported by experimental data in transgenic mice (35–37).

## Clinical Trials Using Senolytics

The enthusiasm for senescent cell clearance across diseases of aging, along with a therapeutic approach that could be translated to patients, led to the first clinical trial of senolytic therapy (D+Q) in adults with idiopathic pulmonary fibrosis (IPF). The intermittent treatment strategy was well-tolerated and improved physical function (gait speed) (105). A second Phase I study in adults with diabetic kidney disease reported a D+Q dependent clearance of senescent adipose and skin cells and reduced SASP within 11 days of treatment (106). Using the safety and feasibility data from these first-inhuman trials demonstrating that intermittent D+Q was well tolerated in 50 subjects (NCT02874989 and NCT02848131), combined with the efficacy data in mouse models, we initiated a Phase I study of senolytic therapy in older adults with early-stage symptomatic AD, SToMP-AD (Senolytic Therapy to Modulate the Progression of Alzheimer’s Disease, NCT04063124) (107).

### Phase I Trial: D+Q in Early Alzheimer’s Disease, SToMP-AD

The primary objective of the vanguard SToMP-AD trial was to evaluate CNS penetrance of D and Q and collect data relevant to safety of intermittent orally delivered senolytic therapy. The secondary endpoints included collecting data on cognition, neuroimaging, and plasma and cerebrospinal fluid (CSF) biomarkers before and after treatment. The intermittent treatment design follows preclinical evidence that demonstrated senescent cells require 2–4 weeks to develop and complete senescent cell clearance is not required for disease modifying effects. In tau transgenic mice we saw improvements in brain structure and function by reducing senescent cell burden by 30% with an intermittent treatment strategy (35), which is consistent with preclinical studies on other models of aging and/or disease (37, 45, 46, 101–103, 108–112). D, a chemotherapy, is a powerful drug and can have serious side effects. However, the intermittent, not continuous, administration of senolytics reduces concerns of toxicity, and effectively circumvents potential off-target effects due to continuous receptor occupancy or modulation of an enzyme or biochemical pathway. Given that the Phase I clinical trial in IPF demonstrated a reduction of senescent cells and SASP within eleven days after a single treatment of D+Q, and improvements in physiological outcomes after 10 weeks (105), we reasoned that a three-month period would provide important feasibility, compliance and efficacy data, as well as potentially reveal other cognitive and biomarker effects useful for designing a larger study.

A total of five participants (mean age: 76±5 years; 40% female) completed the SToMP-AD vanguard trial. To determine CNS penetrance, the levels of D and Q in CSF were measured using high-performance liquid chromatography with tandem mass spectrometry. Safety evaluation involved continuous monitoring and reporting of adverse events, as well as tracking vitals and laboratory work. The treatment increased levels of D and Q in the blood for all participants. In CSF, D levels were detected in four participants (80%) with a CSF to plasma ratio ranging from 0.422% to 0.919%, while Q was not detected. The treatment was well-tolerated, with no early discontinuations. Six mild to moderate adverse events occurred during the study, but no serious events were reported. Cognitive and neuroimaging endpoints did not show significant differences between baseline and post-treatment. Analysis of CSF biomarkers revealed that IL-6 and GFAP levels increased from baseline to post-treatment, coinciding with a trending decrease in cytokines and chemokines associated with senescence. Additionally, there was a trend toward higher (beneficial) levels of Aβ42 (99). Thus, while the study was not specifically designed to assess efficacy, the data indicate potential treatment-related changes in markers associated with cellular senescence and AD pathology.

As other Phase I studies using D+Q in the context of AD report out, we will be positioned to begin determining whether there is a convergence of data for specific disease states (e.g., NCT04785300 and NCT05422885 are both AD Phase I trials using D+Q), or study/site-specific changes are noted. For example, participants with chronic kidney disease reported changes in IL-1α, IL-6, and MMPs-9 and −12 (106), while we observed changes in IL-6, TARC/CCL17, IL-17A, I-TAC/CXCL11, Eotaxin-2/CCL24, Eotaxin/CCL11, and MIP-1a in our AD study participants (99). The low CSF-to-plasma ratio of compound D and the absence of compound Q in CSF may suggest the need for Phase Ib dosing studies to explore novel strategies for increasing Q bioavailability and D levels in the CNS. Nevertheless, given that clearing senescent cells in peripheral tissues has systemic benefits, including the reduction of systemic inflammation, we cannot rule out the possibility that D+Q in the periphery may positively impact the brain (113). While we advise caution in interpreting the secondary and exploratory outcomes data from Phase I trials, our initial study demonstrated the safety and tolerability of compounds D and Q in older adults with AD (99). Collectively the prior work support moving forward with a Phase II, placebo-controlled study of D+Q (NCT04685590).

### Phase II SToMP-AD

**Figure 3.**
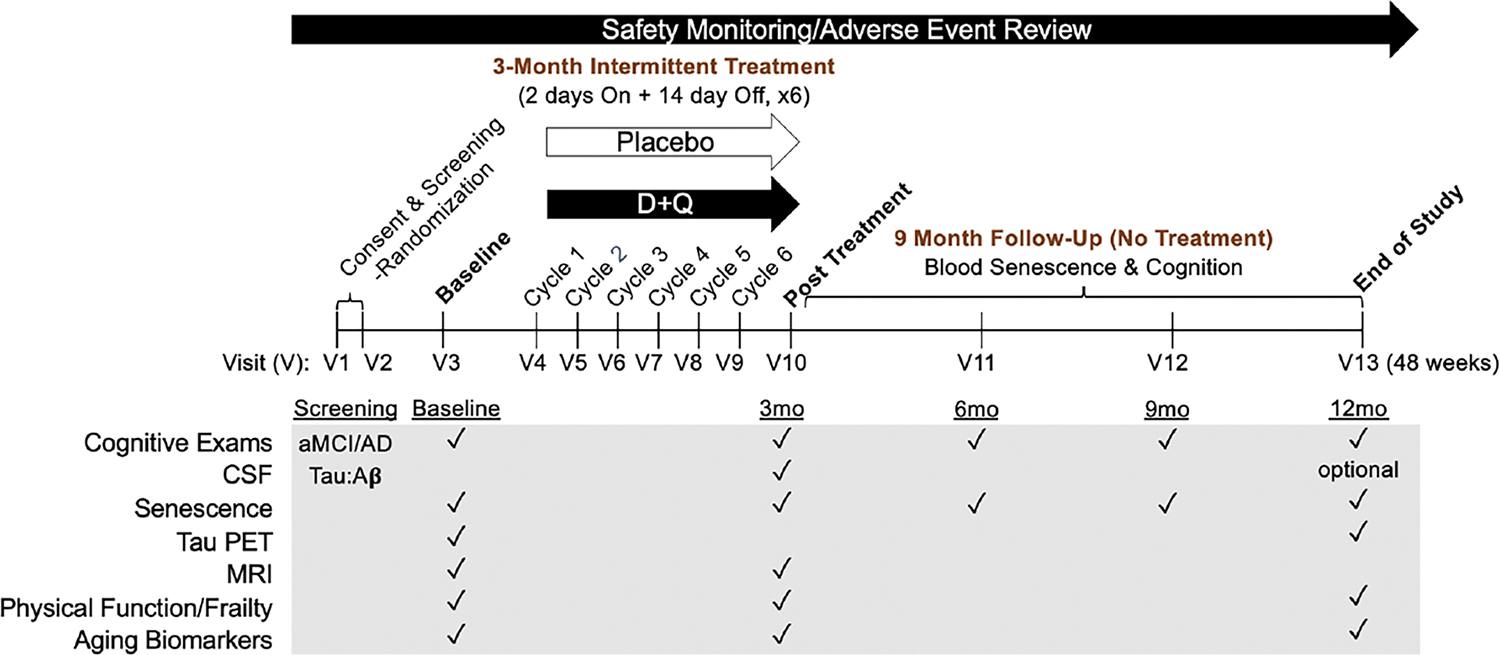
Outline of the Phase II SToMP-AD study timeline Major measures collected at each study visit (V) are indicated by black checkmarks. Abbreviations: D: dasatinib; Q: quercetin; V: visit; CSF: cerebrospinal fluid; PET: positron emission tomography; MRI: magnetic resonance imaging; aMCI: amnestic mild cognitive impairment; AD: Alzheimer’s disease; mo: months.

To rigorously test D+Q in the context of AD, we have initiated a Phase II multi-site, randomized, double-blind placebo-controlled trial (Phase II SToMP-AD, NCT04685590). The primary objective is to determine safety through adverse event (AE) monitoring, vitals, and laboratory assessments. Forty-eight participants, ≥60-years old, with amnestic MCI or early AD (CDR=0.5 or 1; NIA-Alzheimer’s Association criteria) and elevated tau protein, as determined by CSF Aβ:tau ratio, are eligible (Table 2). The study is powered to determine target engagement of senolytics in peripheral blood based on data generated from a Phase I D+Q trial in IPF (106). Participants are randomized 1:1 to receive D+Q or placebo administered for two consecutive days out of every fifteen days across three months with an extended nine-month follow-up after the intervention. The 12-month trial design enables us to assess senolytic-associated changes before and after treatment during the 3-month active treatment phase, track the reappearance of senescent cells after their removal during the 9-month post-treatment period, and evaluate disease trajectory differences over a one-year period (Figure 3). Our primary outcome is change in senescent cell burden in blood as measured by CD3+ p16^INK4A+^ T cells and SASP factors. Plasma will be assessed pre- and post-treatment (baseline and three months) as well as every three months during the 9-month no-treatment follow-up period. We will also collect necessary data to evaluate target engagement in the CNS and disease-modifying effects in aMCI/early AD. CNS target engagement will be assessed by measuring levels of key senescence markers in CSF at baseline and after three months treatment with senolytics. As such, secondary outcomes include changes in cognition, disease severity and function before and after treatment and across the one-year study period and measures of senescence and AD biomarkers in CSF pre- and post-treatment (baseline and three months) with an optional end of study collection. We are also collecting data on brain structure and function through magnetic resonance imaging (MRI) and tau deposition as assessed by tau positron emission tomography (PET) changes between baseline and end-of-study. Although we may not have sufficient power to detect significant changes in our secondary outcomes (tau PET, MRIs, CSF AD biomarkers and cognition), including a nine-month follow-up will help provide preliminary evidence of successful modification of disease trajectory over a one-year period. The Wake Forest University School of Medicine Alzheimer’s Disease Research Center is coordinating the study, which is sponsored by the Alzheimer’s Drug Discovery Foundation (ADDF). The study is anticipated to complete in 2025.

## Summary: Current Limitations and Optimism for the Future

Recent FDA-approved amyloid lowering therapies provide modest clinical effects. Considering their safety concerns and high costs, a significant need to identify additional treatment options remains. Advanced chronological age stands alone as the single common, and greatest, risk factor for developing AD. Regardless of genetic predisposition, lived experiences and disease heterogeneity, the risk of developing AD unvaryingly increases with age across the global population. Biological aging interventions, in isolation and/or in combination, hold potential for preventing, delaying and treating AD and other age-associated conditions. The “geroscience hypothesis” proposes this principle but necessitates experimental testing. Given the extensive experimental data demonstrating health benefits from senescent cell clearance and significant advances in cellular senescence drug discovery, senolytics have emerged as a leading translational geroscience therapy.

The SToMP-AD trial will test the geroscience hypothesis by investigating the impact of targeting cellular senescence, a secondary hallmark of aging, on AD pathogenesis and other biological aging hallmarks. The trial endpoints include both traditional AD outcomes (e.g., cognition, brain imaging, AD fluid biomarkers) and gold standard aging measures (e.g., frailty, physical function, epigenetic age, etc.). While the ultimate goal of the geroscience approach is to extend healthy lifespan by delaying or preventing disease, the lack of specific biomarkers for senescent cells (fluid, imaging, etc.) represents a major hurdle for identifying participants that may most benefit from senolytics. For example, in our evaluation of postmortem human brain tissue from individuals with various levels of AD neuropathology, we observed a wide range (0–15%) of senescent cell burden (39). The current limited knowledge of senescent cell burden and heterogeneity across ages, disease stages or ethnicities compels early trials, such as ours, to enroll symptomatic participants. This strategy is needed not only to provide foundational evidence for the safety and efficacy of senescent cell removal, but also to begin collecting much needed biomarker data that could be used as surrogate markers for senescent cell identification and tracking in pre-clinical/prodromal disease stages. For example, the neuroscience field has identified senescent cells in multiple CNS diseases and disorders, and emerging data highlight their critical importance in driving disease phenotypes. Specifically, beyond amyloid and tau, defective autophagy (114, 115); insulin resistance/obesity (110, 116); traumatic brain injury (TBI) (117); depression (118); alcohol use disorder (119), Parkinson’s disease (120) and pain (121) all reportedly increase CNS senescent cell burden. Given the epidemiology linking each of these independently to increased AD risk, targeting senescent cells in early disease stages (preclinical and/or prodromal) may hold promise to prevent chronic neurodegeneration and dementia.

Senescent cell biomarkers are also critical for designing senolytic therapies. Many molecules involved in establishing the senescence cell fate also play critical roles in cell survival or apoptosis pathways. Developing senolytics with high selectivity and efficacy in targeting senescent cells while minimizing off-target effects on healthy cells remains an ongoing challenge. In fact, since D+Q targets a range of biological pathways in diverse tissues, it is generally difficult to attribute cell type-specific biological effects to the senolysis itself (45, 103, 112). In response to the challenges faced, multiple approaches, pathways, and strategies are under investigation e.g., senolytics and senomorphics. With the increasing interest in senescent cell removal or manipulation, a working group to align study outcomes may be beneficial to better understand efficacy and, potentially, begin considering combination therapies for the treatment of ADRD and other CNS diseases. Given the early stages of this therapeutic approach senescence therapeutics will need to overcome challenges related to biomarker identification, target engagement, CNS penetrance and safety. The AD field successfully overcame similar challenges with amyloid lowering strategies; we are optimistic that AD researchers and trialists will apply the same tenacity and lessons learned to senolytics and other promising biology of aging interventions. Ultimately, we anticipate that the continued development of precise and effective senotherapeutic interventions will lead to the targeted elimination of senescent cells, their detrimental SASP, and improved health outcomes in age-related diseases such as AD.

## Figures and Tables

**Table 1.** Senescent Cell Clearance Improves Outcomes in AD Mouse Models

AD Pathology	Mouse Model	Senescent Cell Types	Senolytics
Tauopathy (NFTs, neurodegeneration, brain atrophy, inflammation, cognitive dysfunction)	rTg(tau_P301L_)4510 *Mapt*^0/0^ rTg(tau_P301L_)4510 rTg(tau_WT_)21221	Neurons	D+Q (35)
	PS19 PS19-INK ATTAC	Microglia & Astrocytes	Navitoclax (ABT263) & AP20187 (36)
Aβ (Aβ plaques, inflammation, cognitive dysfunction)	Tg(APPswe, PSEN dE9)	Oligodendrocyte precursor cells	D+Q (37)

**Table 2.** Inclusion and exclusion criteria for the SToMP-AD Phase II Trial

Inclusion Criteria	Exclusion Criteria
Age 60 years and older at study entry, both sexes, all ethnicities	Body mass index (BMI) > 40 kg/m2
Clinical diagnosis of aMCI or early AD as defined by: aMCI: o CDR = 0.5, Memory domain score ≥0.5; o MMSE 24–30 o WMS-R Logical Memory II <11 for ≥ 16 years education, ≤ 9 for 8–15 years education, ≤ 6 for 0=7 years education Early AD: o CDR 0.5 or 1.0 o MMSE 20–30 o WMS-R Logical Memory II ≤ 8 for ≥ 16 years education, ≤ 4 for 8–15 years education, ≤ 2 for 0–7 years education	Chronic heart failure or QTcF ≥ 450 msec in males and ≥ 460 msec in females
Elevated tau protein, as determined by CSF Aβ:tau ratio	Magnetic Resonance Imaging (MRI) contraindications
Ability to provide written consent or be accompanied by a Legally Authorized Representative (LAR)	Any significant neurologic disease other than prodromal or early AD
Availability of a study partner who agrees to attend all study visits and has at least 10 hours of contact with the participant a week	Current or history of alcohol or substance abuse or dependence within the past 2 years
Absence of travel plans that would interfere with scheduling visits over the 12 months of study duration	Pregnancy or possible pregnancy
Normal blood cell counts, coagulation panel, liver and renal function without clinically significant excursions. Total cholesterol <240 mg/dl, HbA1c ≤ 7%. PT/PTT/INR within normal limits.	Uncontrolled diabetes (HbA1c >7% or the current use of insulin or sulfonylureas)
FDA-approved medications for AD (e.g., donepezil, rivastigmine, galantamine) are permitted if a stable dose has been maintained for ≥3 months prior to study entry	Poorly controlled BP (systolic BP > 160, diastolic BP > 90 mmHg)
	eGFR < 10 ml/min/1.73 m2
	Myocardial infarction, angina, stroke, or transient ischemic attack in the past 6 months
	Presence of significant liver disease with total bilirubin > 2X upper limit
	Anticoagulants other than low dose Aspirin, unless able to be held for 2 days prior to LP and with the documented approval of the prescribing clinician
	Medications that are sensitive to substrates or substrates with a narrow therapeutic range for CYP3A4, CYP2C8, CYP2C9, or CYP2D6 or strong inhibitors or inducers of CYP3A4 (e.g., cyclosporine, tacrolimus, or sirolimus)
	Current medications that induce cellular senescence (i.e. alkylating agents, anthra-cyclines, platins, other chemotherapy)
	Active cancer treatment within last year
	Inability to tolerate oral medication
	H2 antagonists or proton pump inhibitors who are unable or unwilling to reduce or hold therapy for at least 2 days prior to and during each of the 2-day courses of Dasatinib plus quercetin dosing. Instead, subjects may use antacids prior to and during each of the 2-day courses of Dasatinib plus quercetin dosing
	Co-enrollment in another ADRD research study with a potentially disease-modifying intervention or study drug that may impact senescent cells. Participants previously enrolled in a study meeting these criteria are eligible to screen after a washout period of ≥6 months from date of last dose to date of screening
	Presence of any condition that the Investigator believes would put the subject at risk or would preclude the patient from successfully completing all aspects of the trial

## Glossary:

Amyloid hypothesis: proposes that the accumulation of amyloid-beta protein in the brain is the primary cause of Alzheimer’s disease, leading to the formation of plaques and subsequent neurodegeneration
CAR-T Therapy: Chimeric antigen receptor (CAR) T-cell therapy refers to an immunotherapeutic approach aimed at harnessing the anti-cancer potential of T cells. Through *ex vivo* genetic modification, T cells are engineered to express chimeric antigen receptors, enabling them to recognize and eliminate cancer cells with enhanced specificity and efficacy
Cholinergic hypothesis: the degeneration of cholinergic neurons and reduced acetylcholine levels in the brain play a critical role in the development of AD
Frailty: A medical and geriatric syndrome characterized by a decline in physiological reserves and increased vulnerability to stressors, leading to an increased risk of adverse health outcomes
Hallmarks of aging: A concept in the field of biology and gerontology that refers to a set of interconnected cellular and molecular processes and characteristics associated with the aging process. These hallmarks represent the underlying biological mechanisms that contribute to the aging of living organisms. The concept was initially proposed in 2013 (13) and was updated in 2023 (14)
Healthspan: The portion of life spent in good health and functional independence in which an individual remains free from significant disease, illnesses or chronic health conditions that substantially impact daily living
Inflammatory hypothesis: Chronic inflammation, particularly in the brain, plays a significant role in the development and progression of various age-related diseases and neurodegenerative conditions
Intermittent Treatment: Administration of a therapeutic intervention at specific intervals rather than continuously. The approach involves alternating periods of treatment and non-treatment
MCI: Mild Cognitive Impairment refers to a condition that falls between the normal cognitive changes associated with aging and more severe cognitive decline seen in dementia. It is characterized by noticeable and measurable cognitive deficits that are greater than typical age-related changes but not severe enough to significantly interfere with daily activities or independence. Amnestic MCI (aMCI) primarily affects memory, and individuals may have difficulty remembering recent conversations, appointments, or events
Mitochondrial cascade hypothesis: Mitochondrial dysfunction and the accumulation of reactive oxygen species lead to neuronal damage and ultimately contribute to the pathogenesis of neurodegenerative diseases, particularly AD
Progeroid: A condition where young organisms appear much older than their chronological age/premature aging
Proteostasis: maintenance of protein homeostasis in cells, involving processes that regulate protein synthesis, folding, degradation, and clearance to ensure proper protein function and prevent cellular dysfunction and disease
SASP: The senescence-associated secretory phenotype refers to molecules released by senescent cells that both help them survive, and that signal immune cell clearance. SASP factors include cytokines, chemokines, extracellular remodeling proteins
SCAP: Senescent-cell anti-apoptotic pathways engaged by senescent cells to ensure survival/avoid apoptosis
Senolytics: A class of therapeutics that induce senescent cell death by targeting their pro-survival pathways
Senomorphics: A class of therapeutics that reduce the toxicity of senescent cells by dampening their secretome (e.g., SASP)
Senotherapeutics: Term referring to the general therapeutic strategy of targeting and/or disrupting senescent cells
Tauopathy: A group of neurodegenerative diseases that arise concomitant with abnormal tau protein deposition in brain cells

## Abbreviations:

Aβ: Amyloid beta
AD: Alzheimer’s disease
ADDF: Alzheimer’s Drug Discovery Foundation
ADRD: Alzheimer’s disease and related dementias
CDR: Clinical Dementia Rating
CNS: Central nervous system
CSF: Cerebrospinal fluid
COX-2: Cyclooxygenase 2
D: Dasatinib
D+Q: Senolytic therapy including the combination of Dasatinib and Quercetin
FDA: Food and Drug Administration
FOXO4: Forkhead box protein O4
FOXO4-DRI: FOXO4-D-Retro-Inverso
PET: Positron emission tomography
MAPK: Mitogen-activated protein kinas
MDM2: Murine double minute 2
mTOR: Mammalian target of rapamycin
MRI: Magnetic resonance imaging
NFT: Neurofibrillary tangle
OPC: Oligodendrocyte precursor cell
PI3Kδ: Phosphatidylinositol 3-kinase-delta
PL: Piperlongumine
Q: Quercetin
ROS: Reactive oxygen species
SToMP-AD: Senolytic Therapy to Modulate the Progression of Alzheimer’s Disease, acronym for clinical trial
USP7: Ubiquitin-specific peptidase 7
TBI: Traumatic brain injury
uPAR: Urokinase-type plasminogen activator receptor
WAT: White adipose tissue
